# Unraveling the Function of PPARα in Neurodegenerative Disorders: A Potential Pathway to Novel Therapies

**DOI:** 10.3390/biomedicines13112813

**Published:** 2025-11-18

**Authors:** Ourania-Natalia Galanou, Maria Konstandi

**Affiliations:** Department of Pharmacology, Faculty of Medicine, School of Health Sciences, University of Ioannina, 451 10 Ioannina, Greece; orianagalanou22@gmail.com

**Keywords:** Alzheimer’s disease, Parkinson’s disease, neurodegenerative disorders, PPARα

## Abstract

Alzheimer’s (AD) and Parkinson’s (PD) diseases are the most prevalent neurodegenerative disorders (NDs), posing a growing global health burden due to the lack of effective therapies. Current treatments offer only limited symptomatic relief without preventing the progression of NDs. In the search for novel therapeutic strategies, peroxisome proliferator-activated receptor alpha (PPARα) has emerged as a promising therapeutic target because mounting evidence suggests that PPARα activation can effectively modify key pathological mechanisms related to NDs, including neuroinflammation, mitochondrial dysfunction, oxidative stress, and impaired transcriptional regulation, processes leading to protein misfolding and aggregation. This review focuses on the potential therapeutic relevance of PPARα activation in AD and PD, discussing mainly insights from preclinical studies. Indicatively, gemfibrozil (PPARα agonist) markedly reduced the beta-amyloid burden, microgliosis, and astrogliosis in the hippocampus of 5xFAD mice and ameliorated their spatial learning and memory. Fenofibrate (PPARα agonist) reduced the depressive-like behavior and memory deficits in rotenone-lesioned rats developing Parkinsonism. It also restricted the depletion of striatal dopamine and protected their substantia nigra pars compacta from dopaminergic neuronal death and α-synuclein aggregation. Clinical trials gave disparate results, indicating either a benefit of fenofibrate in cognitive decline of AD patients or limited efficacy. The role of PPARα agonists in PD is less well established in human trials, which provided limited evidence of neuroprotection and reduced neuroinflammation. Although current findings are promising, they underscore the necessity of further rigorous clinical validation of the efficacy of various PPARα agonists in the retardation or even prevention of AD and PD symptomatology in both genders and the development of reliable biomarkers for the early assessment of the impact of PPARα agonists on NDs. The safety of these drugs in the elderly and their longitudinal effectiveness should also be evaluated.

## 1. Introduction

Alzheimer’s (AD) and Parkinson’s (PD) diseases are the most common age-related neurodegenerative disorders (NDs), which constitute a critical health concern worldwide, because of the increased life expectancy [1,2]. The World Health Organization (WHO) reported in March 2025 that 57 million people had dementia worldwide, with AD accounting for 60–70% of all cases and PD following as the second-most common neurodegenerative disease [3].

**Figure 1:**
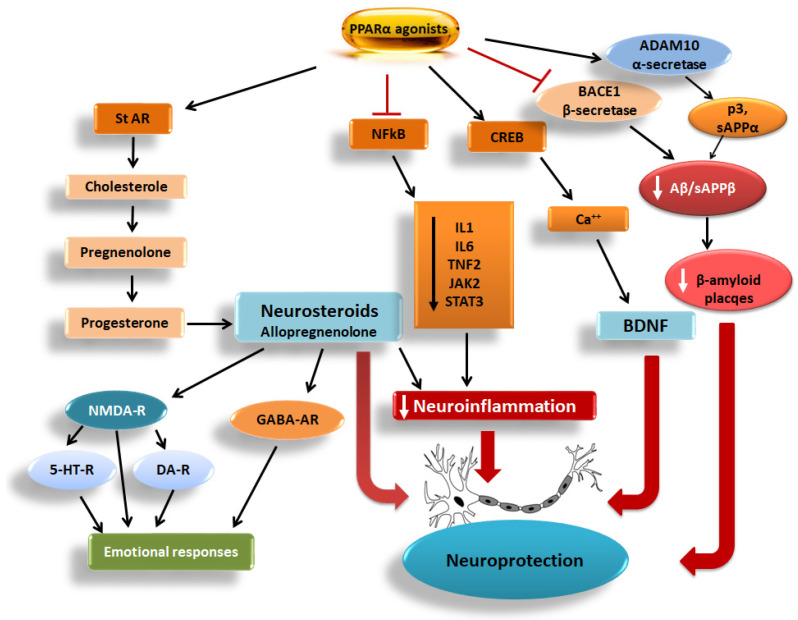
Pathways related to the PPARα activation-induced neuroprotection, including the neuroactive steroids, the CREB/Ca++/BDNF pathway, the NFkB signaling mediating neuroinflammation, and the BACE1/sAPPβ/Aβ-mediated Aβ plaque formation. StAR: steroidogenic acute regulatory protein, CREB: cAMP-response element binding protein, BDNF: brain-derived neurotrophic factor, NFkB: nuclear factor kappa-light-chain-enhancer of activated B cells, IL1: interleukin 1, IL6: interleukin 6, TNF2: tumor necrosis factor-alpha promoter variant 2, JAK-2: Janus kinase 2, STAT3: signal transducer and activator of transcription 3, Ca++: calcium ions. BACE1: β-secretase, ADAM10: α-secretase, sAPPβ/α: soluble amyloid precursor protein β and α, Aβ: beta-amyloid protein, p3: p3 peptide, NMDA-R: N-methyl-D-aspartate receptor, GABA-AR: gamma-aminobutyric acid type A receptors, DA-R: dopaminergic receptors, 5-HT-R: serotonergic receptors, according to Roy et al. [8]. Modified from Żulińska et al. [9]. Flat-headed red arrows depict blockade and white/black arrows in the boxes/ellipse depict reduction, whereas heavy red arrows depict induction.

**Figure 2:**
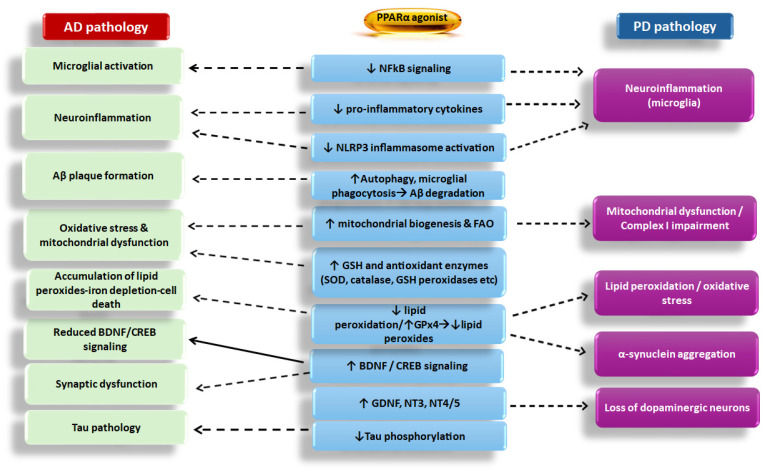
Effect of PPARα agonists on Alzheimer’s disease (AD) and Parkinson’s disease (PD) pathologies and the main mechanisms implicated. NFkB: nuclear factor kappa-light-chain-enhancer of activated B cells; NLRP3: NOD-, LRR-, and pyrin domain-containing protein 3 inflammasome; CREB: cAMP-response element binding protein; BDNF: brain-derived neurotrophic factor; Aβ: beta-amyloid peptides; GSH: glutathione; FAO: free fatty acid oxidation; SOD: superoxide dismutase; GPx4: glutathione peroxidase 4; GDNF: glial cell-derived neurotrophic factor; NT3 and NT4/5: neurotrophins 3 and 4/5. The dotted arrow depicts inhibition, whereas the black arrow depicts restoration/stimulation. Upward arrows in the boxes depict induction/increase, whereas downward arrows depict inhibition/reduction.

Overall, from a pathophysiological point of view, several genetic, environmental, and endogenous factors hold key roles in the pathogenesis of NDs [4]. Furthermore, processes inducing deregulation of transcription may lead to protein aggregation and misfolding, thus eventually provoking apoptosis and, consequently, exacerbation of the age-related neuronal loss [4,5]. However, it should be noted that all known processes that are implicated in the development of NDs, including chronic inflammation, microglial activation, oxidative stress, and neuronal mitochondrial impairment, simultaneously affect each other along a continuum rather than as separate and isolated linear metabolic events that affect specific points of the neuronal processing and cerebral function [6]. In this context, it is of interest to note that there is a bidirectional relationship between protein aggregation and inflammation, where each process exacerbates the other, rather than following a simple linear path. In this bidirectional loop, peroxisome proliferator-activated receptor α (PPARα) and its coactivator peroxisome proliferator-activated receptor gamma coactivator 1 alpha (PGC1α) can interfere by inhibiting the nuclear factor kappa-light-chain-enhancer of activated B cells (NFkB)-inflammatory pathway (Figure 1 and Figure 2). This indicates that the PGC1α/PPARα axis can act as a cellular defense mechanism against the damaging cycle of protein aggregation and inflammation [7].

This pathophysiology is a fundamental hallmark of these NDs and is associated with the dysfunction of neural synapses and the deposition of altered variants of proteins in the brain [8]. In this line, in AD, neurons of the hippocampus and entorhinal cortex are primarily degenerated, followed by neurons in cortical areas. This gradual neuronal degeneration leads to cognitive decline in advanced disease stages. In contrast, the neuronal damage in PD initiates in extranigral areas, such as the olfactory bulb and the brainstem, before evolving into the substantia nigra and later spreading into the entire neocortex, thus inducing both motor and cognitive symptomatology [8].

To date, the therapeutic management of AD and PD focuses on the alleviation of the symptoms or the retardation of their progression [9]. Among the challenges the clinicians face in the treatment of their patients is the permeative potency of most prescribed drugs through the blood–brain barrier (BBB), which in most cases is low [3]. The fact that the available therapies provide only symptomatic relief to the patients also underscores the urgent necessity for the design and development of neuroprotective medications with improved efficacy and toxicity profiles.

Currently, increased attention has been drawn to the therapeutic potential of selective peroxisome proliferator-activated receptor (PPAR) agonists [10]. PPARs belong to the superfamily of nuclear receptors and work as ligand-activated transcription factors. PPARα, PPARβ/δ, and PPARγ are included in this family of receptors that normally contribute to the homeostasis of cellular metabolism. In this context, they have been involved in several metabolic pathologies, as they control glucose and lipid metabolism and regulate inflammatory genes [10,11].

PPARα is the only isoform colocalizing with all cell types (neurons, astrocytes, and microglia) in various adult mouse and human brain tissues, which implies that PPARα may have predominant roles in cerebral functions depending on the cell type [12]. Notably, studies using AD and PD murine models provided evidence suggesting gender differentiation in *Pparα* expression in their brains. Age-related differences were also revealed [12].

It is well established that PPARα regulates the energy homeostasis of the body by controlling lipid and glucose metabolism, the redox state, and the glutamatergic cholinergic/dopaminergic neurotransmission in the brain. Deregulation of these mechanisms is known to underlie the development of NDs [13]. PPARα is also engaged in the metabolism of amyloid precursor protein (APP) in the brain, where it may directly or indirectly affect Tau protein phosphorylation via beta-amyloid proteins (Aβ) [13,14].

Experimental studies using an AD mouse model demonstrated that PPARα activation can protect the neurons by maintaining mitochondrial integrity, ensuring protein homeostasis, and promoting neuronal repair mechanisms like axonal growth and remyelination [15].

PPARα agonists also induced the synthesis of the brain-derived neurotrophic factor (BDNF) and its receptor tropomyosin receptor kinase B (TrkB) (Figure 1 and Figure 2), thus favoring neuroprotection [15,16,17,18]. The anti-inflammatory properties of PPARα agonists are well demonstrated in several experimental models of inflammation [14], further supporting the notion that these drugs should be considered as good candidates to be clinically validated for the treatment of NDs. To date, the majority of data supporting this hypothesis comes from experimental studies employing in vitro and animal models of AD and PD, which apparently cannot be directly translated to the human condition.

In the current review, findings coming mainly from preclinical and some preliminary clinical studies investigating the effectiveness of PPARα agonists on NDs were summarized because, to our knowledge, there are only a few, still ongoing, relevant clinical trials. Therefore, robust clinical trials are essential to assess the efficacy of various PPARα agonists in the retardation or even prevention of the symptomatology of the distinct NDs in both genders.

## 2. Effect of PPARα on Pathophysiological Features of Neurodegenerative Disorders

### 2.1. PPARα Signaling

PPARα acts as a nuclear receptor and transcription factor that shows gender-specific patterns, with higher expression levels in male T cells, suggesting different immune responses between genders [19,20]. Interestingly, reduced *Pparα* expression levels were observed in the brains of AD mouse models at ages where cognitive deficits emerge, indicating an age-dependent decline in PPARα function [21].

The PPARα-linked signaling pathway is crucial for managing lipid β-oxidation, mitochondrial metabolism, and energy homeostasis, particularly in the liver, heart, muscle, and brain [14,22]. It also regulates neuroinflammation in the central nervous system (CNS) and holds a key role in the homeostasis of redox state and in the glutamatergic, cholinergic/dopaminergic neurotransmission [14].

PPARα responds to fatty acids and drugs like fibrates to regulate target gene expression. Using a double immunofluorescence assay, it was found that only PPARα is expressed in all cell types of the adult human and mouse brain. Specifically, PPARα was detected in various hippocampal regions of mice, including the CA1, CA2, and CA3, and in their dentate gyrus [23].

PPARα, as PPARβ/δ and PPARγ, has five functional domains: the N-terminal domain (A/B) with a ligand-independent transactivation function 1 (AF-1) and a mitogen-activated protein kinase (MAPK) phosphorylation site, the DNA-binding domain (DBD) with two zinc fingers that bind to peroxisome proliferator response elements (PPREs), the flexible hinge region (E), the ligand-binding domain (LBD), and finally, the C-terminal domain with another ligand-dependent activation domain (AF-2), which is crucial for the binding of coactivators [23,24]. Upon activation with fatty acids, fibrate drugs, or other agonists, PPARα forms heterodimers within the nucleus with the retinoid X receptor (RXR) [24]. The binding of a ligand to this nuclear receptor induces conformational changes, releases corepressors, and recruits a coactivator complex, including the cyclic AMP-response-element-binding protein (CREB)-binding protein (CBP) and histone acetyltransferase p300 [24]. PGC1α is also a coactivator that enhances the ability of PPARα to activate the transcription of genes implicated in fatty acid oxidation and mitochondrial function [25]. Along with the coactivators, the PPARα/RXR heterodimer binds to PPRE, and the histone acetyltransferase is activated, resulting in the transcription of target genes involved in both glucose and lipid metabolism, as well as in the control of inflammation [24]. Polyunsaturated fatty acids are the preferred endogenous PPARα ligands, but also, various lipids, such as saturated fatty acids, fatty acyl-CoA species, prostaglandins, leukotrienes, oxidized fatty acids, and phospholipids, are also considered PPARα activators [26]. Specifically, PPARα activation increases fatty acid oxidation by upregulating genes, that encode enzymes that break down fatty acids for energy, such as carnitine palmitoyl transferase-1 (CPT1) [22,27]. PPARα also modifies lipoprotein metabolism by inducing genes, including Apolipoprotein A-I (ApoA-I), that are involved in high-density lipoprotein (HDL) formation, thus facilitating the removal of cholesterol from tissues [28]. PPARα is also implicated in the cholesterol transport to mitochondria, where it is used as a substrate for neurosteroid biosynthesis [9] (Figure 1). PPARα also promotes fatty acid transport by increasing the expression of proteins involved in fatty acid transport and metabolism, like fatty acid-binding proteins (Fabps) [22]. Furthermore, PPARα activation inhibits inflammation by repressing the interconnected NFkB and activator protein-1 (AP-1)-linked pro-inflammatory signaling pathways [29] (Figure 1 and Figure 2).

### 2.2. Effect of PPARα Activation on Neuroinflammation

Whereas neuroinflammation constitutes a defense mechanism protecting the brain from diverse pathogens, it has been proven that sustained inflammatory responses end up being detrimental to the brain [30,31] because they trigger an immune response within the central nervous system (CNS) involving the hyperactivation of microglia and astrocytes [30], which hold dual roles in NDs. Initially, they aim at the protection of neurons and the maintenance of homeostasis [32]; however, with the progress of disease, these glial cells lead to chronic neuroinflammation and thereby exacerbate the pathology of NDs [32]. In particular, early in NDs, microglia protect the brain by clearing toxic proteins and damaged cells, a process mediated by the tumor necrosis factor (TNFα), interleukins IL1β, IL16, IL18, and the C-C motif chemokine ligand 2 (CCL2) [30,31]. Nonetheless, if the stimulus persists, microglia become overwhelmed or dysfunctional, and their ineffective clearance mechanisms result in chronic inflammation and the production of reactive oxygen species (ROS) that can accelerate the progression of NDs [30]. In addition, in chronic inflammation, the phosphorylation of MAPKs activates several nuclear transcription factors, including NFkB, which leads to the transcription of genes, which further encode inflammatory mediators in neural cells [30]. Within the spectrum of detrimental effects that chronic inflammation induces in the cerebral functions, the disruption of BBB integrity, which permits the entrance of immune and inflammatory cells from the periphery into the brain, leads to activation of glial cells and neurons [30]. Consequently, the classical activation of microglia leads to the release of more pro-inflammatory mediators, nitric oxide (NO), ROS, and proteases, which induce neurodegeneration [30].

It is known that the toll-like receptor (TLR)-NFkB signaling is the canonical “priming” event for NLRP3 inflammasome activation, as it induces the transcription of NLRP3 and pro-IL1β. A second signal (e.g., K+ efflux, mitochondrial ROS, or lysosomal rupture from phagocytosed aggregates) triggers NLRP3 oligomerization, ASC recruitment, caspase-1 activation, and the cleavage of pro-IL1β/IL18 into mature cytokines, thus driving neuroinflammation and pyroptotic-like microglial responses [33].

Various experimental studies supported the neuroprotective role of PPARα. As mentioned before, PPARα holds a key role in modulating inflammatory responses by a mechanism implicating its binding to co-repressors, thus inhibiting key inflammatory signaling pathways, including those linked to NFkB, Janus kinase/signal transducer-activator of transcription (JAK/STAT), and MAPKs that contribute to neuroinflammation [12]. Specifically, PPARα activation suppresses the activity of NFkB, thus inhibiting the transcription of pro-inflammatory genes, including those encoding cytokines (IL1β and IL6), chemokines (CCL2, CCL3, and CXCL10), and the adhesion molecules ICAM-1 and VCAM-1 [22,34,35] (Figure 2).

Experimental studies employing murine cell lines and animal models showed that in microglia and astrocytes, PPARα activation suppressed NFkB signaling, mainly via trans-repression, without requiring direct DNA binding of PPARα. This process implicates direct protein–protein interactions with NFkB subunits. It is also known that PPARα activation affects NFkB by influencing the stability of its inhibitor, IkBα, and modulating several upstream signaling pathways. Activation of PPARα can prevent the translocation of the NFkB p65 subunit from the cytoplasm to the nucleus, thereby inhibiting its ability to act as a transcription factor [36] (Figure 2). Furthermore, PPARα can directly inhibit the activity of other transcription factors, such as AP-1, without directly binding to DNA via a “trans-repression” mechanism, interfering with the inflammatory signaling cascade [37]. In addition to the aforementioned mechanisms, PPARα activation can indirectly dampen inflammatory signals by upregulating enzymes that catalyze fatty acid oxidation (FAO), thus preventing the accumulation of fatty acids that trigger inflammation [38].

Based on these anti-inflammatory effects, PPARα activation could alleviate chronic neuroinflammation via repression of NFkB transcriptional activity, the subsequent down-regulation of pro-inflammatory cytokines [22,34,35] and NLRP3 inflammasome, thus effectively reducing the priming signal from TLR activation. It was also suggested that PPARα agonists, by increasing mitochondrial biogenesis and the synthesis of antioxidant enzymes, such as GPx4, reduced lipid peroxidation and lysosomal destabilization from aggregated lipids, thus restricting the activation of NLRP3 [39] (Figure 2).

An in vitro study using SIM-A9 microglial cells reported that PPARα endogenous agonists, such as N-acylethanolamine (NAE) derivatives of fatty acids [N-palmitoylethanolamine (PEA), N-oleoylethanolamine (OEA), N-arachidonoylethanolamine (AEA), and N-docosahexaenoylethanolamine (DHEA)], managed to effectively suppress the lipopolysaccharide (LPS)-mediated neuroinflammation and glial activation by suppressing the protein expression of pro-inflammatory cytokines (IFNγ, TNFα, IL1β, IL6). Furthermore, an in vivo study employing male C57BL/6 mice, submitted to the y-maze and novel object recognition tests, indicated that the above-mentioned NAE derivatives reversed the harmful effects of LPS-induced neuroinflammation on hippocampal neurogenesis, spatial working memory, and long-term memory functions [40] (Table 1). Notably, in several preclinical studies using various models of PD and AD, exogenous PEA administration appeared to have neuroprotective effects that are potentially mediated via the PEA-induced PPARα activation, which prevents the overexpression of NFkB, IL1β, and TNFα, thus reducing neuroinflammation (Table 1). However, the neuroprotective effects of the above-mentioned drugs are not exclusively PPARα-dependent, as other receptors and mechanisms may also contribute to these effects, including those related to PPARγ, PPARβ/δ, cannabinoid, and transient receptor potential V1 (TRPV1) receptors [41,42,43].

Apart from the direct role of PPARα in neuroinflammation, the PPARα-related neuroprotective mechanisms are equally important, because PPARα holds a central role in microglial activation and in astrocyte function [61,62]. Specifically, PPARα activation reduced the overactivation of astrocytes and microglia, indicating that PPARα agonists could reduce reactive gliosis. It should be noted that PPARα activation has the potential to directly shift microglia from the pro-inflammatory M1 state to the anti-inflammatory/pro-repair M2 state, resulting in a higher release of BDNF and other neurotrophic factors and a lesser secretion of IL1β/IL6/TNFα [63] (Figure 1 and Figure 2). The role of PPARα activation in microglia shift from state M1 to M2 is well defined in animal models, because in the absence of PPARα signaling, macrophages are unable to either appropriately suppress cytokine production or acquire an oxidative metabolic program related to the M2 phenotype [64]. Nonetheless, while animal studies provided strong evidence for the role of PPARα, further research is essential to confirm these findings in humans and fully understand the physiological role of PPARα in regulating microglial function, because PPARα interacts with and influences numerous other signaling pathways that are potentially involved in the regulation of microglial activation. To date, the precise interplay between PPARα and these complex regulatory networks is not fully mapped out.

On the other hand, the neuroprotection of astrocytes is based mainly on their key role in the metabolic support of neurons by supplying them with the essential nutrients. They also maintain the integrity of the BBB while regulating lactate and glutamate levels in the brain [65,66]. Specifically, astrocytes facilitate the clearance of excess glutamate from the synapses, thus preventing excitotoxicity, which is characterized by neuronal damage and death [65].

Glutamate is the predominant excitatory neurotransmitter in the CNS, holding a crucial role in synaptic plasticity, neuronal signaling, and cognitive functions. Astrocytes maintain glutamate homeostasis in the CNS by glutamate uptake. Glutamate transporter-1 (GLT-1/EAAT2) is the primary glutamate transporter responsible for 90% of forebrain glutamate uptake in the adult CNS and prevents excitotoxicity by removing excessive glutamate from the extracellular space [67]. Notably, it was found that GLT-1/EAAT2 concentration is markedly decreased in NDs. Studies that have used transgenic mice expressing the human APP gene bearing the APP London mutation found in familial AD have indicated reduced expression of GLT-1/EAAT1 and EAAT2 in their neocortex [68]. It was found that there is a GLT-1 dysfunction in the brains of AD patients involving reduced expression and function of GLT-1, which has been associated with exacerbation of their cognitive deficits and pathology. Further investigation revealed that there is a complex interplay between the impaired PPARα signaling observed in AD patients that contributes to GLT-1 loss in the hippocampus and cerebral cortex and the overall disease progression [69]. It is of interest to note that PPARα activation increased kynurenic acid (KYNA) production, a metabolite of tryptophan and N-methyl-D-aspartate (NMDA) antagonist that displays neuroprotective properties and modulates glutamate signaling via GLT-1/EAAT2. It was suggested that PPARα activation could prevent the internalization of GLT-1/EAAT2 in astrocytes via inhibition of the PKC-linked signaling pathway, thus increasing the amount of available GLT-1/EAAT2 on the cell membranes to clear the excessive glutamate from the synaptic cleft and thereby reduce excitotoxicity [68,70,71]. However, another study indicated that PPARα activation reduced GLT-1/EAAT2 protein levels in the membranes of astrocytes by 40%, an effect potentially indicating enhanced endocytosis of GLT-1 receptors in cortical astrocytes that might attenuate the clearance of extracellular glutamate molecules, thus raising the risk of neuro-excitotoxicity induced by the excessive amount of glutamate molecules at synapses [70]. These contradictory findings suggest that whereas some PPARα agonists may display beneficial effects in certain contexts, their potential to negatively affect GLT-1/EAAT2 function in clearing the excessive glutamate from synapses could pose a therapeutic risk. Therefore, the overall impact of PPARα activation on NDs needs to be carefully assessed.

Astrocytes can undergo a process known as reactive astrogliosis due to excessive inflammatory cell mediators and oxidative stress [66,72]. In AD, astrocytes can produce Aβ proteins and fail to clear them, thus leading to their accumulation in the brain [73]. As a result, dysfunctional astrocytes can fail to regulate the extracellular environment of the brain, leading to increased oxidative stress, excitotoxicity, and disrupted BBB, conditions that all accelerate neurodegeneration [66]. This detrimental process is also present in PD, including the presence of reactive astrocytes in the substantia nigra pars compacta (SNpc) [66], which creates a self-perpetuating cycle where the presence of inflammatory mediators from both hyperactivated microglia and reactive astrocytes sustains inflammation, prevents resolution, and can contribute to neuronal dysfunction and neurodegeneration [66].

Studies using experimental models of AD and PD revealed that PPARα activation influenced astrocyte metabolic support to neurons. In particular, PPARα activation in astrocytes appeared to promote metabolic support by enhancing glutamate uptake and degradation of Aβ peptides while also contributing to mitochondrial function and reducing inflammation in ND models [29,74] (Figure 1 and Figure 2).

The central role of PPARα in neurosteroid production in the brain is also important. It was reported that PPARα agonists induced the synthesis of neurosteroids in mitochondria, which, along with the circulating steroids produced in peripheral organs and readily penetrate the BBB, significantly restricting neuroinflammation in the mouse brain [29] (Figure 1 and Figure 2).

### 2.3. Effect of PPARα Activation on Misfolded Proteins

The pathophysiological mechanisms of NDs also include the formation of misfolded proteins that build up and aggregate within neurons to form inclusions, tangles, or plaques [8]. AD is characterized by Tau tangles and Aβ plaques, whereas PD is characterized by the accumulation of α-synuclein in Lewy bodies [8,75].

In AD, excessive accumulation of Aβ peptides is the main constituent of extracellular amyloid plaques [76]. These peptides are derivatives of the APP and are produced through proteolysis by β- and γ-secretases [76]. Hyperphosphorylated Τau proteins, forming intracellular neurofibrillary tangles, are also implicated in the etiopathology of AD [77].

Specifically, the formation of Aβ peptide-containing plaques in the brain is connected to neurofibrillary tangles (NFTs) made of hyperphosphorylated Tau [78]. Oligomers of different structures are created when Aβ monomers group together and combine to create Aβ fibers, which form Aβ plaques, a process resulting in the gradual conversion of healthy neurons to diseased ones, an effect that is mediated by the Aβ plaque-triggered inflammatory response [78]. Excessive presence of diseased neurons potentiates inflammation, thus leading to extensive neuronal loss, an effect that results in altered brain function and cognitive decline [78,79,80,81].

In this context, the potential role of PPARα in the therapy of AD was assessed [59] according to its role in the progressive deposition of Aβ proteins in the brain parenchyma [82] or in the mutation of α-synuclein that leads to Aβ misfolding and aggregation [78], processes that trigger the activation of glial and other immune/inflammatory cells [78], which in turn, release inflammatory cytokines, chemokines, and various neurotoxic substances that induce neurodegeneration within the substantia nigra [78,79,80,81].

Several experimental studies indicated that PPARα signaling regulates proteostasis by repressing protein synthesis and secretion while promoting protein degradation, including Aβ peptides, through pathways like autophagy (a cellular “clean-up” process that removes damaged proteins and organelles) and proteasomal degradation (Figure 2). PPARα signaling through interaction with other signaling pathways, like that of heat shock factor-1 (HSF-1), also contributes to stress resilience and can suppress protein aggregation [83,84].

A current experimental study employing adult A53T mice reported that treadmill exercise and fenofibrate decreased α-synuclein aggregation in nigral neurons and promoted its clearance via PPARα activation, which upregulated lysosomal biogenesis, known for its determinant role in the clearance of the misfolded α-synuclein that drives neurodegeneration in PD and related NDs. In particular, oral administration of fenofibrate inhibited α-synuclein spreading by ~50% and protected nigral dopaminergic neurons in the preformed fibrils (PFF)-seeded A53T mouse brain. The central role of PPARα in α-synuclein regulation was apparent, because no similar effects were observed in the brains of *Pparα*-deficient mice [55] (Table 1). It was also reported that PPARα activation by low doses of gemfibrozil induced lysosomal biogenesis and autophagy. The drug also increased the uptake and degradation of Aβ peptides in astrocytes of 5xFAD mice (AD model) [84] (Table 1).

PPARα activation also appeared to reduce misfolded proteins in iPSC-derived neurons by enhancing protein clearance pathways like autophagy and by shifting APP processing away from the amyloidogenic pathway. It appears that PPARα activation potentially promotes the degradation of misfolded proteins via autophagy and by upregulating ADAM10, which increases the non-amyloidogenic processing of APP, thus reducing the production of toxic Aβ peptides [85].

### 2.4. Effect of PPARα Activation on Mitochondrial Dysfunction

The mitochondrial antioxidant defense system includes several enzymatic and non-enzymatic antioxidants that neutralize ROS and prevent oxidative damage in cellular structures. It is known that ROS-regulated cell signaling is critical for the progression of NDs, where mitochondrial dysfunction is a common feature, leading to neuronal energy deficits [54,78]. Specifically, the imbalance between the production of ROS and the ability of the brain to detoxify these reactive intermediates leads to cellular damage [66]. Several studies indicated that PPARα participates in the defense mechanisms of the brain, protecting neurons from oxidative injury [86]. This notion is based on the finding that PPARα activation in the CNS reduces oxidative stress by inducing the expression of antioxidant enzymes, thus preventing neuronal damage [11]. In particular, PPARα agonists, including WT-14643, induced the expression of antioxidant enzymes that directly neutralized ROS, such as catalase (CAT) and copper/zinc superoxide dismutase (SOD), and activated the nuclear factor erythroid 2-related factor (2Nrf2) pathway, which leads to increased production of antioxidant proteins like heme oxygenase-1 (HO-1) and uncoupling proteins (UCP2 and UCP3) that clear ROS and reduce oxidative stress [57] (Table 1). It was also found that in mice treated with the PPARα agonist GW7647, the content of intracellular ROS in the liver, measured using a fluorescent probe DCFH-DA, was markedly reduced [87] (Table 1).

In the regulatory pathway of ROS production, the presence of the PPAR triad (PPARα, PPARβ/δ, PPARγ) in the brain holds a central regulatory role via two distinct feedback loops: (a) a positive correlation exists between PPARβ/δ and PPARγ, and (b) a negative correlation exists between PPARα and PPARβ/δ. As mentioned before, PPAR activation suppresses ROS production in the brain by either activating ROS-degrading pathways linked to CAT, SOD, γ-glutamylcystein ligase, glutathione peroxidase 4 (GPx4), and glutathione or by inhibiting ROS-producing pathways [88] (Figure 2). The array of factors related to PPARs includes the Poly(ADP-ribose) polymerase 1 (PARP-1), a DNA-bound enzyme that works alongside PPARα as a mediator of mitochondrial homeostasis. More specifically, PARP1 holds a key role in the epigenetic regulation of nuclear genes involved in mtDNA repair and transcription. In this context, their involvement in mitochondrial dynamics is closely related to the onset and progression of NDs [13].

Several studies support, though, that PPARα activation in the brain can both induce and inhibit ROS, depending on the context and the specific signaling pathways involved. For instance, in some experimental models of retinal or brain ischemia, PPARα activation appeared to reduce ROS production by repressing genes like the NADPH oxidase-4 (*Nox4*) [44]. Notably, PPARα activation effectively prevented the radiation-induced increases in TNFα, IL1β, and Cox-2 in murine BV-2 microglial cells, in part, by inhibiting the NFkB- and AP-1-linked pro-inflammatory pathways, but it did not prevent the radiation-induced intracellular ROS production [37] (Figure 1). Interestingly, in some injury models, PPARα activation has been associated with increased ROS production, though the precise mechanisms involved are still under investigation, as several complex interactions with other signaling pathways may be implicated [89]. It appears that the role of PPARα in ROS production is not a simple “on/off” switch. It is a complex relationship, where the net effect of PPARα activation on ROS production is tissue- and condition-specific, depending on the interplay with other signaling pathways, including inflammation and metabolic function. Overall, PPARα activation can either induce or inhibit ROS production depending on the cellular and molecular context. Therefore, the preservation of the balance between the PPAR-dependent positive and negative pathways is essential for the maintenance of ROS production at normal levels [88].

Given the fact that mitochondrial dysfunction is a common feature in aging, as well as in the progression of NDs, the support of mitochondrial functional integrity is included in the therapeutic goals of these diseases [90]. In the investigation of new therapeutic approaches for NDs, PPARα agonists hold a central role because PPARα activation regulates lipoprotein metabolism and fatty acid β-oxidation, processes that potentiate mitochondrial energy dynamics [74,91]. In particular, PPARα agonists reduce the accumulation of harmful lipid peroxides in the mitochondria of AD and PD animal models that promote ferroptosis and cell death [92]. They regulate lipid metabolism by inducing the expression of enzymes, including carnitine palmityl transferase 1 (CPT1), medium-chain acyl-CoA dehydrogenase, acyl-CoA oxidase (ACOX), and fatty acyl-CoA synthase, thus facilitating energy production from fats. It should be noted also that PPARα activation induces the transcription of genes, which encode transport proteins, so that fatty acids can enter the β-oxidation pathway [93], which is crucial for neuronal function and membrane integrity [13].

Mitochondrial biogenesis is a critical parameter in the regulation of mitochondrial quantity, response to cellular damage, cellular turnover, and energy supply. The regulation of this process is controlled mainly by PPARα and its coactivator, PGC1α [70]; the nuclear respiratory factors (NRF1, NRF2) [70,94] and the mitochondrial transcription factor A (TFAM). These factors have been detected at markedly lower levels in the hippocampus of AD compared to controls, indicating abnormal mitochondrial biogenesis rates in AD [95].

Overall, mounting evidence based mainly on preclinical studies employing in vitro and animal models of NDs suggests that PPARα activation enhances mitochondrial function mainly by promoting fatty acid oxidation and by inducing mitochondrial biogenesis. These PPARα-induced effects are mediated by an increased number and capacity of mitochondria, which are essential for the preservation of energy production, the control of ROS synthesis and elimination, and finally, the cellular health in several tissues, including the brain [96]. It should be noted, though, that there are some conflicting results regarding the effect of PPARα agonists on mitochondrial function, as some studies indicated a protective role by improving mitochondrial respiration and biogenesis, while others suggested that PPARα activation may result in mitochondrial dysfunction leading to apoptosis and endoplasmic reticulum stress, or that its role depends on the cell type and specific stressor [97]. Furthermore, the combined PPARα and PPARγ activation may negatively impact mitochondrial function. This dual activation led to PGC1α repression and lower mitochondrial abundance in the cardiac muscle [98].

## 3. Effects of PPARα Agonists on Neural Plasticity and Cognitive Functions

It is of particular interest to note that there is emerging evidence suggesting that PPARα may hold important roles in cognitive functions, as it is distributed in several hippocampal regions of the brain, indicating its potential involvement in hippocampal functions [86]. It was also reported that PPARα modulates the expression of genes involved in synaptic plasticity and long-term potentiation (LTP) through activation of CREB, a transcription factor regulating genes with determinant roles in learning and memory [86]. The role of PPARα appears to be central because, despite the presence of normal levels of PPARβ/δ and PPARγ, either *Pparα*-null hippocampus or isolated *Pparα*-null hippocampal neurons have deficient calcium influx and lower expression of various plasticity-related molecules (NMDAR2A, NMDAR2B, GluR1, activity-regulated cytoskeleton-associated protein). Notably, the *Pparα*-deficient mice appeared to have weaker spatial learning and long-term memory when compared to wild-type mice [86].

In an experimental transgenic mouse model with cognitive impairment, it was found that PPARα activation improved hippocampal synaptic plasticity via RXR stimulation [99]. In spite of the intestinal PPARα-mediated induction of a noradrenergic transmission in the basolateral amygdala, which facilitates the retention of spatial memory [100], the main and direct effect on spatial memory is attributed to hippocampal PPARα, because its absence inhibits the process of learning and memory acquisition through inhibition of *Creb* transcription and the subsequent repression of various memory-related genes [86].

An experimental study indicated that oleuropein (OLE), the main constituent of *Olea europea* leaf extracts, activated PPARα in neurons and astrocytes of wild-type mice, providing neuroprotection against noxious biological reactions that are induced following cerebral ischemia [17]. It is also of note that chronic per os treatment with OLE up-regulated BDNF and its receptor TrkB in the prefrontal cortex (PFC) of mice, an effect mediated by PPARα activation, because there were no similar modifications in the PFC of *Pparα*-null mice following chronic OLE treatment [17] (Table 1). These beneficial effects of OLE should be potentially attributed mainly to its major metabolite hydroxytyrosol (HT), because OLE pharmacokinetics is characterized by poor bioavailability, rapid hydrolysis to hydroxytyrosol and elenolic acid, and extensive metabolism via liver enzymes and gut microbiota. Whereas OLE itself is only minimally absorbed, HT is more readily absorbed [101]. Despite its rapid metabolism, HT can penetrate the BBB and reach the brain in small but functional amounts to regulate mitochondrial biogenesis, autophagy, redox balance, and inflammation, among others [102]. These neuroprotective effects of HT are improved by other polyphenolic compounds of *Olea europea* [103] and are potentially mediated at least in part by PPARα activation [104]. This study also reported that fenofibrate, a selective PPARα agonist, up-regulated BDNF and neurotrophin-3 (NT3) in the PFC of mice, and it induced NT4/5 expression in their PFC and hippocampus [17] (Table 1). The upregulating effect of PPARα agonists on BDNF is highly significant because this neurotrophic factor is one of the essential regulators of neurotransmission, remyelination, and neural plasticity [17,18,19,31]. BDNF regulates the cellular signaling underlying cognition and, specifically, synaptic efficacy, a determining parameter in learning and memory [17]. BDNF also attenuates memory deficits associated with amyloid-β peptide plaque formation [17,105]. However, it should be noted here that the role of excessive BDNF levels in AD is multifaceted, and its impact varies depending on the patient’s disease stage and individual variability. Whereas BDNF in principle displays neuroprotective properties by supporting neuronal survival and growth, its excessive elevation in AD can coincide with neuroinflammation that may lead to the elimination of its protective effects. Elevated BDNF can also stimulate glutamate signaling, thus leading to excitotoxicity, in particular in NDs, where neuronal health is already compromised. Finally, it appears that the brain’s response to BDNF is modified in NDs, where an excessive increase in this neurotrophic factor could be detrimental [106].

Overall, of particular interest is the accumulating evidence coming from human studies indicating reduced PPARα expression in the brains of AD patients, which is considered to be associated with the main pathological features of the disease, including neuroinflammation, impaired fatty acid transport, mitochondrial dysfunction, and decreased clearance of Aβ plaques. However, the precise mechanism of PPARα signaling alterations in AD remains blurred. The findings that PPARα activation in animal models improves cognitive functions and reduces Aβ pathology by promoting autophagy and stimulating Aβ proteolysis underscore the necessity of thorough clinical validation of the effectiveness of PPARα agonists on AD [13].

## 4. Role of PPARα Agonists in the Treatment of Neurodegenerative Disorders

Various strategies are employed for the therapeutic management of NDs, which either focus on the pathophysiology of these diseases and try to alleviate their symptoms or retard their progression [10]. A critical obstacle in the treatment of NDs is the BBB, which makes the delivery of sufficient therapeutic drug doses to the brain challenging. It appears that the nature of the BBB, along with the low permeability of most drugs, contributes to the lack of effective treatment options for NDs [11].

Currently, increased attention has been drawn to the therapeutic potential of selective PPAR agonists [10]. The three isoforms of this family, PPARα, PPARβ/δ, and PPARγ, normally contribute to the homeostasis of cellular metabolism, and in this context, they have been involved in several metabolic pathologies [11], as they control glucose and lipid metabolism and regulate inflammatory genes [11].

Current experimental studies are shedding light on the potential benefits of PPAR agonists in the treatment of NDs [10,14,35,107]. They indicated that novel highly selective PPAR agonists may have higher efficacy and fewer adverse effects than the traditional drugs used in the treatment of NDs [20,108]. Therefore, in the framework of an innovative therapeutic approach, PPARs could be assessed as therapeutic targets for the development of drugs to prevent or retard neurodegeneration [109].

This review highlights findings from recent studies focusing on PPARα activation for the treatment of AD and PD. While PPARα is mainly known for its role in lipid oxidation in peripheral tissues, including the liver, kidney, cardiac and skeletal muscles, it is also expressed in brain regions, such as the hippocampus, prefrontal cortex, olfactory bulbs, and cerebellum, in particular in cerebellar granule neurons, astrocytes, and microglia [17,108,110]. The role of PPARα in brain functions is currently under intense investigation using predominantly experimental models of NDs. In this framework, it is worth noting that fenofibrate, a PPARα agonist, preserved hippocampal neurogenesis and inhibited microglial activation in wild-type mice following whole-brain irradiation, an effect though not observed in *Pparα*-null mice (Table 1). Thorough investigation confirmed that PPARα was essential for the neuroprotective and anti-inflammatory effects of fenofibrate [51]. The latter effects were attributed to the fenofibrate-induced suppression of cytokine production through the modulation of NFkB signaling [111] (Figure 1).

To date, all available information in terms of the effects of PPARα agonists on AD and PD symptomatology focuses on neurons and astrocytes and not on their effects on the BBB. The impact of PPARα agonists on BBB functions is crucial because this barrier limits the entrance of several substances into the brain [112] and ensures oxygen/glucose delivery and the removal of different metabolic byproducts, thus helping in the maintenance of brain homeostasis [113]. Furthermore, the functional integrity of the BBB is essential for the preservation of the precise neuronal balance of ion gradients that are required to allow the electrical communication between neurons [114,115]. It is worth noting that the neurovascular dysfunction and cerebral amyloid angiopathy of AD patients were associated with the accumulation of Aβ peptides around their cerebral blood vessels, leading to disturbance in BBB integrity [115,116,117]. The apparent relationship between Aβ peptide accumulation in the brain interstitial fluid and BBB damage, though, does not depend only on Aβ production rate but also on Aβ clearance from the brain, suggesting a significant role of Aβ-related transporters [118]. In this context, several experimental studies supported a potentially beneficial role of PPARα agonists in BBB functioning. Specifically, fenofibrate (PPARα agonist) or rosiglitazone (PPARγ agonist) attenuated the disrupted permeability across the BBB induced by the HIV-1-specific protein Tat [52], and WY-14643 (a selective PPARα agonist) attenuated the BBB breakdown during traumatic brain injury in vitro [119] (Table 1). Despite the low BBB permeability of fenofibrate, pretreatment of mice with the drug managed to maintain the integrity of their BBB during cerebral ischemia–reperfusion injury and protected their brains by antioxidant and anti-inflammatory mechanisms, effects that were PPARα-dependent, as they were not apparent in *Pparα*-deficient mice [120].

Taken together, these preclinical data, despite the lack of proven clinical benefit of PPARα agonists in NDs, support the notion that they should be validated in clinical trials as potential novel therapeutic approaches for AD and PD.

### 4.1. PPARα Agonists in the Treatment of Alzheimer’s Disease

Distinct mechanisms have been suggested to address the therapeutic role of PPARα agonists in AD. The related studies have focused mainly on the role of PPARα in maintaining the brain’s energy state through modulation of ketogenesis to provide an alternative fuel source [21,121,122]. It was reported that PPARα agonists stimulated hepatic fatty acid oxidation and ketogenesis in both mice and humans. Specifically, PPARα activation upregulated, among other genes, the *Hmgcs2* encoding the 3-hydroxy-3-methyl-glutaryl-CoA synthase 2 (HMG-CoA synthase 2), a rate-limiting enzyme in ketogenesis that catalyzes the conversion of acetyl-CoA into aceto-acetyl-CoA, the precursor of ketone bodies, which have the capacity to protect hippocampal neurons from Aβ toxicity [21,121,122,123,124].

Another mechanism suggests the involvement of PPARα in mitochondrial functions. It appears that PPARα plays a key role in the regulation of Aβ plaque formation, and it might also be partly involved in the synthesis of BDNF in the hippocampus [125]. Activation of PPARα also contributes to Aβ clearance via the repression of APP and neuronal autophagy, along with reduced Tau phosphorylation [126].

Tau-related kinases are enzymes that, in excess, can cause hyperphosphorylation of Tau proteins, leading to the formation of neurofibrillary tangles associated with AD. Key tau-related kinases include glycogen synthase kinase-3 beta (GSK3β), Cyclin-dependent kinase 5 (CDK5), and MAPKs like ERK2 and JNK. Whereas normal phosphorylation is essential for Tau function, excessive phosphorylation can disrupt microtubules, cause damage in neurons, and thus contribute to AD pathology [126]. Regarding the role of PPARα in the activation of kinases and phosphatases implicated in Tau phosphorylation, there is an intricate interplay where GSK3α promotes PPARα signaling, whereas PPARα activation can inhibit GSK3β activity [127]. However, there is only an indirect regulatory relationship between PPARα and Tau phosphatases [128]. It is considered that the PPARα activation-reduced neuroinflammation in some contexts could be an indirect mechanism for protecting against Tau pathology [128] (Figure 2). The finding that PPARα downregulation contributes to mitochondrial dysfunction in the brain by disrupting fatty acid metabolism, reducing antioxidant defense, and impairing mitochondrial biogenesis and function suggests a link between metabolic control and cellular health that could be relevant to Tau pathology [21].

Notably, PPARα activates α-secretase (ADAM10) and induces the synthesis of the cytoprotective peptides, p3 and sAPPα, that repress the synthesis of Aβ peptides [125]. More specifically, PPARα agonists, including gemfibrozil, increased *ADAM10* expression in hippocampal neurons of mice, an effect not evident in *Pparα*-null mice, redirecting APP metabolism toward the α-secretase and away from the β-secretase (BACE1)/amyloidogenic pathway, thus reducing the synthesis of Aβ peptides [85] (Table 1). These findings are in line with those of another study indicating that PPARα activation in human-derived neuronal cultures increased α-secretase activity and decreased that of β/γ-secretases, a fact that shifts APP processing away from generating Aβ peptides [129] (Figure 1).

The potential therapeutic role of gemfibrozil in NDs has been extensively studied. The drug is used to lower the levels of triglycerides and reduce the risk of coronary heart disease [124]. Preclinical studies employing in vitro and in vivo models indicated that gemfibrozil, by penetrating the BBB efficiently, could retard or even prevent the progression of NDs. It is of interest to note that in the 5xFAD mice, a familial AD murine model, gemfibrozil reduced the Aβ burden, microgliosis, and astrogliosis and eventually ameliorated spatial learning and memory [56,84] (Table 1). Specifically, the drug reduced the number of Aβ plaques and the amount of Thioflavin S-positive area in the hippocampus of 5xFAD mice by 50% [56]. Gemfibrozil also improved the memory of these transgenic mice that carry five mutations linked to AD and lead to rapid amyloid pathology. It is suggested that the gemfibrozil-induced effects are potentially mediated by activation of the PI3k-linked signaling pathway [130]. Interestingly, the drug upregulated IκBα, an anti-inflammatory molecule that inhibits NFkB signaling. Gemfibrozil also appears to regulate several mitochondrial pro-survival factors in the brain by modulating the expression of PGC1α, transcription factor A mitochondrial (TFAM), and nuclear respiratory factor 1 (NRF-1). The significant role of PPARα in cognitive functions is supported by the fact that in the absence of PPARα, no signs of memory improvement were detected in 5xFAD/PPARα^−/−^ mice treated with gemfibrozil [56].

Whereas gemfibrozil is known as a PPARα activator, its effects in the brain are complex. Some of them require PPARα activation, but others proceed via alternative pathways. In particular, high doses of gemfibrozil can induce anti-inflammatory effects in microglia via mechanisms involving mainly PPARβ. They stimulate the DNA binding of transcription factors, such as the glial factor-1 (GF-1), independently of PPARα [131].

To our knowledge, there are several ongoing clinical trials of Phase I/II investigating the potential beneficial effects of gemfibrozil in AD administered at approved doses. In humans, gemfibrozil is generally well-tolerated when administered at the dose of 600 mg per os x2/day that is used against dyslipidemias, with only common mild side effects and less common severe risks, including myopathy, rhabdomyolysis, bone marrow depression, and pancreatitis [132]. To date, these ongoing clinical trials have indicated the anti-amyloidogenic properties of fibrates in AD patients. Interestingly, these patients had lower Aβ_1–42_ peptide levels when compared to age-related, untreated healthy controls [133] (Table 1).

Similarly, bezafibrate, a pan-agonist for all PPAR isoforms, improved cognitive functions and reduced neuroinflammation by enhancing mitochondrial biogenesis and oxidative phosphorylation through the PPAR-PGC1α pathway [134]. It is of interest also to note that a new pan-PPAR agonist, GFT1803, decreased Aβ plaques and microglial activation in a murine AD model by promoting Aβ clearance via an insulin-degrading enzyme (IDE)-mediated mechanism [135]. Nonetheless, we should bear in mind that whereas PPAR pan-agonists have the potential for improved efficacy by combining the benefits of selective PPAR agonists, they also have an increased toxicity risk, including hepatocarcinogenesis, in particular, when administered at high doses long-term. The goal of current research focuses on finding a balance, through the development of selective or partial PPAR agonists and modulators, in order to achieve optimal therapeutic benefits with minimum off-target effects and toxicity [136].

In vitro experiments employing APPsw/SH-SY5Y cells exposed to Aβ peptides demonstrated that the PPARα agonist GW7647 enhanced the transcription of GPx4 and decreased the iron transport compared to controls. GW7647 also induced *Pgc1α*, *Nrf2*, and *Tfam* in female AD Tg mice bearing the “London” mutation in APP, an effect of paramount significance, because these genes encode proteins participating in mitochondrial biogenesis [58] (Table 1). A study employing the murine AD APP/PS1 model demonstrated that GW7647 inhibited β-secretase (BACE1), thus lowering the synthesis of Aβ peptides (Table 1). Apart from reducing Aβ burden, this PPARα agonist also diminished lipid peroxidation and inflammation and improved ameliorated cognition [35]. More specifically, GW7647 induced the expression of the antioxidant enzyme GPx4, an indirect index of the reduction in iron deposition and oxidative stress. Taking into consideration the aforementioned findings, it appears that activation of the PPARα/PGC1α/NRF2/TFAM pathway and mtDNA biosynthesis at an early stage of AD could be an effective method to retard or even postpone the pathology of AD [58].

It is of particular interest that the PPARα activation-mediated neuroprotection can be strengthened by PPARγ activation, because PPARγ activates the Bcl2 anti-apoptotic proteins and improves mitochondrial activity, antioxidative processes, and neuronal survival [137,138]. Specifically, the combined activation of PPARα and PPARγ is followed by suppression of macrophage and microglia activation, thus preventing inflammatory cells from entering the CNS, an effect that protects the brain from the aggravation of inflammatory processes that trigger neurodegeneration and neuronal death [139].

In conclusion, accumulating evidence based mainly on preclinical studies suggests that PPAR-based therapies appear to be promising in the treatment of AD when compared with other treatment options, including the anti-amyloid and the anti-tau therapies. PPARα agonists appear to offer a path toward the creation of innovative and effective therapeutic interventions in AD because they target receptors involved in the synthesis of factors affecting various pathological features of the disease [90].

To date, clinical trials for AD have assessed the effectiveness of several PPAR agonists as a therapeutic target, particularly PPARγ agonists like thiazolidinediones, mainly because of their anti-inflammatory and anti-amyloidogenic properties. While the findings coming from preclinical studies that have employed animal models of AD are promising, relative clinical trials have shown variable results, with some PPAR agonists demonstrating limited efficacy and significant adverse effects, including edema and several cardiac manifestations. Current research aims to identify selective PPAR agonists, in particular, PPARα and dual PPARδ/γ, penetrating the BBB with fewer side effects. Despite past setbacks, the broad spectrum of beneficial effects of PPARα agonists in AD pathology strongly supports the continuation of their clinical validation [140].

### 4.2. PPARα Agonists in the Treatment of Parkinson’s Disease

Parkinson’s disease is a chronic and progressive age-related ND that affects up to 3% of the global population over 65 years of age. In 2021, approximately 11.77 million people worldwide had the disease, with the number estimated to be more than double, up to 25.2 million, by 2050 [141]. This progressive ND has as its central manifestations the impairment of motor and cognitive functions [5]. Numerous genetic factors are implicated in the evolution of PD, including mutations in several genes (ubiquitin C-terminal hydrolase, α-synuclein, leucine-rich repeat kinase 2, Parkin RBR E3 ubiquitin-protein ligase, PTEN-induced kinase 1, protein deglycase, and glucocerebrosidase), among others [142]. Several environmental factors are also incriminated, like exposure to pesticides, methanol, and carbon monoxide poisoning [142]. The pathological manifestations of PD occur when there is an important loss of dopaminergic neurons, mostly in the substantia nigra, which, along with the buildup of α-synuclein in brainstem neurons, results in the formation of Lewy bodies, a histopathological hallmark of the disease [143,144,145,146].

While PD therapy usually focuses on the motor deficits due to the loss of nigrostriatal dopaminergic neurons, the non-motor symptoms, mainly cognitive and emotional, which are provoked by both a forebrain cholinergic neuronal loss and an increased cholinergic tone in the striatum, also need to be addressed effectively [147,148,149].

In this context, several studies recognized that PPAR agonists may protect nerve cells from oxidative stress, inflammation, and programmed cell death in NDs, including PD [142,149]. In particular, there is direct experimental evidence suggesting that PPARα activation reduced the dopaminergic neuron firing in the Ventral Tegmental Area (VTA) via increased phosphorylation of the β2 subunit of nicotinic acetylcholine receptors (β2nAChRs) [142]. While the SNpc is the primary dopaminergic region affected in PD, the VTA is primarily involved in reward and motivational circuits [150]. PPARα activation with fenofibrate caused a switch from tonic to phasic activity in dopamine cells of the rodent VTA and increased dopamine- and cAMP-regulated phosphoprotein, 32 kDa (DARPP-32) phosphorylation in their nucleus accumbens [151]. Under pathological conditions, such as neuroinflammation or oxidative stress, overactivation of nicotinic receptors in the VTA may lead to excessive dopamine release, mitochondrial stress, and neuronal dysfunction [152]. In this context, PPARα agonists may exert a neuroprotective role by dampening the hyperexcitability of VTA dopaminergic neurons [5,153]. Meanwhile, in the SNpc and striatal circuits, PPARα agonists may support dopaminergic survival and function, indirectly contributing to improved motor outcomes in PD models [5,153]. In this context, PPARα serves as an intrinsic regulator of cholinergic transmission, thus stabilizing dopaminergic tone in PD [5,153] (Figure 2). As mentioned before, PPARα activation also leads to a significant reduction in neuroinflammation, oxidative stress, and mitochondrial dysfunction, all of which are major pathological features of PD [10] (Figure 2).

On the other hand, the neuroprotective role of PPARγ has been previously studied as a treatment option for PD [154], employing in vitro and in vivo animal models. It was found that the PPARγ agonist MDG-548 exhibited neuroprotection in resident microglia reactive to LPS by reducing phagocytosis mediated by STAT6 activation [10]. MDG-548 also induced the production of IL10 via the NFkB pathway, highlighting the role of PPARγ in macrophage differentiation and neuroinflammation [10]. It was also reported that pioglitazone, a PPARγ agonist, maintained mitochondrial membrane potential and reduced mitochondrial ROS production and proliferative-associated microglia (PAM). The drug also decreased the synthesis of inflammatory cytokines and the activation of NFkB [150]. Rosiglitazone, another PPARγ agonist, was found to be protective against mitochondrial dysfunction induced by the complex I inhibitor, MPTP, by increasing mitochondrial membrane potential and the expression of SOD and CAT, along with the expression of Bcl2 and Bax, thus limiting apoptosis and promoting the antioxidant defense [155].

Experimental studies indicated that fibrates (fenofibrate, clofibrate) are the main PPARα agonists displaying a significant neuroprotective effect that is connected mainly with the maintenance of glutamate homeostasis, the cholinergic/dopaminergic communication, along with the reduced pro-inflammatory signals and astrogliosis in the CNS [47,48] (Table 1). Other preclinical studies employing a PD murine model indicated that fenofibrate restricted the dopaminergic neuronal loss in the substantia nigra of PD mice and attenuated the loss of tyrosine hydroxylase immune reactivity in their striatum compared with the untreated PD mice [49]. Fenofibrate (100 mg/kg per os) also decreased hypolocomotion in PD mice 24 h following infusion of their SNpc with MPTP (PD model) and their depression-like behavior 22 days later. The drug also protected their dopaminergic neurons and restricted the production of ROS 23 days following infusion [50]. When fenofibrate (100 mg/kg per os) was administered to rotenone-lesioned rats that developed Parkinsonism, it reduced their depressive-like behavior and memory deficits. Furthermore, fenofibrate reduced the depletion of striatal dopamine and protected their SNpc from dopaminergic neuronal death. The drug also attenuated α-synuclein aggregation in the striatum and SNpc of PD rats [45] (Table 1). These beneficial effects may be due to fenofibric acid, the main active metabolite of fenofibrate in the brain [46]. The dose of fenofibrate used in these experiments is considered high (100 mg/kg per os), involving the risk of side effects. In clinical terms, the highest dose suggested for patients is 145 mg/day, which, in general, is effective in reducing triglycerides and total cholesterol levels while inducing only mild side effects [156].

MHY908, a dual PPARα/γ agonist, demonstrated protective effects in an MPTP mouse PD model by reducing motor deficits, dopaminergic neuronal loss, and astroglial activation in the nigrostriatal pathway of PD mice. The neuroprotection of MHY908 was confirmed using primary astrocytes and appeared to be mediated by inhibition of the NFkB signaling pathway [53,157]. Nonetheless, all MHY908-related evidence is based on preclinical studies, and its translation into humans remains untested.

Similarly, gemfibrozil protected dopaminergic neurons and improved the movement of MPTP-exposed mice by stimulating the astroglial-derived neurotrophic factor (GDNF)-linked pathway [53] (Table 1).

Another PPARα agonist, PEA, demonstrated neuroprotective effects in the MPTP mice model of PD by reducing neuroinflammation, motor deficits, and neuronal damage, potentially via PPARα-dependent pathways [45,158]. There are also several ongoing clinical studies investigating the potential of other PPARα agonists to retard or prevent the progression of PD [45] (Table 1). A small Phase II pilot clinical trial using ultra-micronized PEA (600–1200 mg/day) indicated improvement in memory and attention, as well as in motor and non-motor symptoms of PD patients, with excellent safety and tolerability [159]. There is also a Phase II clinical trial of gemfibrozil (FHL-301, NCT05931484) for early-stage PD patients, investigating its safety, tolerability, and efficacy [60] (Table 1).

In summary, PPARα agonists are promising for the treatment of AD and PD by reducing neuroinflammation, providing neuroprotection, and improving mitochondrial function. They regulate the expression of genes implicated in cellular survival, inflammation, and oxidative stress, displaying specific benefits including improvement of cerebrovascular function and shifting microglial responses to a more protective phenotype. But, whereas they are promising, more robust clinical trials are essential to validate their efficacy and safety in AD and PD. New molecules, PPARα agonists, should also be synthesized with improved properties in order to overcome the limitations of present compounds, including their limited BBB penetration and their considerable systemic side effects, which in turn should be evaluated in well-designed preclinical studies.

## 5. Conclusions

This review elucidates the neuroprotective impact of PPARα agonists, which is based on the multifaceted role of this nuclear receptor in the CNS. It is primarily due to the ability of PPARα agonists to reduce oxidative stress and inflammation, regulate lipid metabolism, improve vascular function, and support neuronal health and repair mechanisms. In this context, PPARα activation may help mitigate some of the key pathological processes that lead to neurodegeneration by restricting the activity of microglia and astrocytes, reducing oxidative stress, and supporting mitochondrial function. All these PPARα-mediated effects make this receptor a promising target for therapeutic interventions aiming at retarding or reversing the progression of NDs. Nonetheless, the looming beneficial effects of PPARα agonists on neurodegenerative disorders are based mainly on promising findings from preclinical studies using cell and animal models of AD and PD. This is a subject of growing interest, but still, there are several gaps in the clinical evidence due to limited well-designed clinical trials in humans, which, in most cases, have focused on metabolic diseases. It is conceivable that the differences between ND models and related human pathologies largely prevent the findings from animal studies from being directly translated to effective human therapies. Another obstacle in the translation of experimental data is the inconsistency in the reproducibility of PPARα findings across the distinct ND models, which is due to several factors, including variations in experimental setups, cell types used, and genetic backgrounds. Another particularly important issue in pharmacological research is the fact that the majority of in vivo studies, in general, employ male experimental animals, and, therefore, comprehensive knowledge about the effectiveness of PPARα agonists on AD and PD pathology in females is lacking.

Although ND manifestations usually appear later in life due to gradual, progressive processes that may begin earlier, for the better understanding of these diseases, in particular in women, it is crucial to include female models in the experimental design, incorporating age and estrous cycle, as the sex-steroid hormonal fluctuations during the cycle or the lack of these hormones can significantly impact disease progression. Employing females in addition to males could improve scientific accuracy by revealing masked gender differences, providing mechanistic insights, and ensuring that sex-steroidal status is taken into account in experimental design.

Finally, the exact mechanisms underlying the PPARα agonist-mediated neuroprotective effects are not fully understood. For instance, whereas a clear role for PPARα in mitochondrial function is supported by several studies, other studies reported conflicting results. Similarly, the effect of PPARα activation on APP processing appears to be model-dependent, with some models indicating an inverse relationship and others a more complex regulatory network. Further research is also needed to elucidate the interaction of PPARα activation with the neuroinflammatory pathways and cellular stress responses in the brain. Targeted clinical studies assessing the efficacy of PPARα agonists across the distinct NDs are also lacking. Furthermore, there is insufficient clinical data on the long-term safety and effectiveness of PPARα agonists in the elderly, and therefore, longitudinal studies are essential to evaluate potential side effects and the sustainability of therapeutic benefits of PPARα agonists in NDs. Limited knowledge also exists about the optimal dosages and formulations of PPARα agonists for neuroprotection in men and women, and therefore, the expected variations in pharmacokinetics and pharmacodynamics across individuals should be the ground for more tailored preventive and therapeutic approaches. In this framework, the development of reliable biomarkers for the assessment of the impact of PPARα agonists in NDs is a challenging but essential task in order to assess treatment efficacy and disease progression.

Addressing these gaps requires a concerted research effort, including well-structured clinical trials, to assess the effectiveness of PPARα agonists in the retardation of the progression and even in the cure of NDs. The potential neuroprotective effects of the combined treatment using PPARα and PPARγ agonists should also be further assessed in AD and PD in the framework of preclinical and clinical studies, emphasizing on the determination of the optimal conditions (dosage and time) in order to avoid the potentially important side effects of the dual treatment.

## Figures and Tables

**Table 1:** Effects of PPARα agonists in clinical trials and experimental models of Alzheimer’s and Parkinson’s disease.

PPARα Agonist	Experimental Model	Main Outcomes	Clinical Trial (Phase & Main Outcomes)
**Oleuropein/Hydroxytyrosol**	129/Sv mice and *Pparα*-null mice, 100 mg/kg/day per os	↑ BDNF and TrkB in PFC [17]	No AD/PD RCTs
**Fenofibrate**	129/Sv mice and *Pparα*-null mice 0.2% in rodent chow; AD murine model (APP/PS1, 100 mg/kg per os); cell cultures; MPTP rat model of PD	↑ BDNF and NT3 in PFC & ↑ NT4/5 in PFC & hippocampus [17]; ↓ Aβ42 and BACE1 by ~40% (APP/PS1); ↓ IL1β and TNFα ~30–50% [12]; ↓ ROS [44]; ↓ inflammation, hypolocomotion & DA neuronal death in SNpc of a PD MPTP rat model [45,46]. Preserved glutamate homeostasis, cholinergic/dopaminergic communication & ↓ α-synuclein in SNpc of a PD model [45,47,48,49,50]. Preserved hippocampal neurogenesis & inhibited microglial activation following whole brain irradiation [51]; ↓ disrupted permeability across BBB [52].	Not tested in large scale AD/PD RCTs
**Gemfibrozil**	MPTP mouse model of PD (C57BL/6 mice, 60 mg/kg/day per os, 7–14 days); 6-OHDA rat model (30–60 mg/kg i.p.)	↑ striatal dopamine ~20–30%; preserved T-helper cells + neurons by ~35%; ↑ astrocytic GDNF release [53]; reduced microglial Iba1 by ~40% [12]; ↑ ADAM10 and ↓ Aβ production [54]; Improved spatial learning & memory [55,56]; ↓ Aβ accumulation and reversed memory deficits & anxiety symptoms [56]	Ongoing Phase I/II AD & Phase II PD clinical trials (NCT05931484). Gemfibrozil ↓ Aβ_1–42_
**WY-14643**	APP/PS1 transgenic mice (AD model) 100 mg/kg/day per os for 2–4 weeks	↓ inflammation markers, ↓ Aβ plaque load (~25–40%), ↓ ROS, ↑ PGC1α & mitochondrial biogenesis [24,57,58]	Research tool compound
**GW7647**	APPswe SH-SY5Y cells (1–10 μM) and APP/PS1 transgenic mice, 3–10 mg/kg/day per os, 2–4 weeks	↑ GPx4 protein 2–3 fold; ↓ lipid peroxidation markers (MDA) 40%. ↓ iron uptake by ~30% [36]; ↓ Aβ synthesis and release; ↓ sAPPβ and BACE1 [59]	Research tool compound
**Palmitoylethanolamide (PEA)**	MPTP mouse PD model and LPS-induced neuroinflammation models (10–30 mg/kg/day per os or i.p., 7–14 days).	↓ TNFα & IL1β 50%; ↓ microglial activation ~40%; ↑ BDNF ~30%; improved motor activity [12]; some cognitive benefit	Phase II pilot RCT (PEA 600–1200 mg/day); memory, attention, motor activity improvement [60]
**Pemafibrate (SPPARMα, a highly specific PPARα agonist)**	Rat cerebral ischemia/reperfusion models following high-fat diet (0.3–1 mg/kg/day per os, 4–8 weeks).	↓ liver & plasma TG 50–70%; ↓ IL-6, MCP-1 (CCL2) 30–40%. ↓ neuroinflammation, ↑ BDNF & mitochondrial biogenesis, improved memory and spatial learning [12]	no AD/PD RCTs

PPARα: peroxisome proliferator activated receptor alpha; AD: Alzheimer’s disease; PD: Parkinson’s disease; RCT: randomized controlled clinical trial; BDNF: brain derived neurotrophic factor; TrkB: tropomyosin receptor kinase B; NT3 and NT4/5: Neurotrophic factor 3 and 4/5; TG: triglycerides; MCP-1: Monocyte chemoattractant protein-1; TNFα: tumor necrosis factor α; IL1β: interleukin 1β; sAPPβ: soluble amyloid precursor protein β; Aβ: β amyloid peptide; APP/PS1: mouse model of Alzheimer’s disease; MDA: Malondialdehyde; BACE1: beta-site amyloid precursor protein cleaving enzyme 1 (β-secretase); GPx4: glutathione peroxidase 4; APPswe: human Alzheimer β-amyloid precursor protein carrying the Swedish mutation; PGC1α: Peroxisome proliferator-activated receptor gamma coactivator 1 alpha; ADAM10: α-secretase; GDNF: Glial cell line-derived neurotrophic factor; MPTP: 1-methyl-4-phenyl-1,2,3,6-tetrahydropyridine (PD model); 6-OHDA: 6-hydroxy-dopamine; PFC: prefrontal cortex; ROS: reactive oxygen species; BBB: blood–brain barrier; DA: dopaminergic; SNpc: substantia nigra pars compacta; The numbers in brackets indicate the corresponding references. Downward arrows depict inhibition/reduction, whereas the upward arrows depict induction/increase.

## Data Availability

This review contains data that are available from the published articles appearing in PubMed.

## Abbreviations

AD	Alzheimer’s disease
PD	Parkinson’s disease
NDs	Νeurodegenerative Disorders
PPARs	Peroxisome proliferator-activated receptors
PPARα	Peroxisome proliferator-activated receptor alpha
PPARγ	Peroxisome proliferator-activated receptor gamma
PPARβ/δ	Peroxisome proliferator-activated receptor β/δ
PAMPs	Pathogen-Associated Molecular Patterns
PARP-1	Poly (ADP-ribose) polymerase 1
DAMPs	Damage-Associated Molecular Patterns
CNS	Central Nervous System
PRRs	Pattern Recognition Receptors
TLRs	Toll-like receptors
TNFα	Tumor necrosis factor α
IL	Interleukin
CCL2	C-C motif chemokine ligand 2
ROS	Reactive Oxygen Species
MAPK	Mitogen-activated protein kinase
NFkB	Nuclear factor kappa-light-chain-enhancer of activated B cells
BBB	Blood-brain-barrier
NO	Nitric oxide
Aβ	Beta-amyloid peptide
BDNF	Brain-derived neurotrophic factor
APP	Amyloid precursor protein
NFTs	Neurofibrillary tangles
UCP2	Uncoupling protein 2
CPT1	Carnitine palmityl transferase 1
JAK/STAT	Janus kinase/signal transducer-activator of transcription
NAE	N-acylethanolamine
LPS	Lipopolysaccharide
PEA	N-palmitoylethanolamine
GLT-1	Glutamate Transporter-1
PKC	Protein kinase C
EAAT2	Excitarory amino acid transporter 2
GLAST/EAAT1	Glutamate transporter/Excitarory amino acid transporter 1
KYNA	Kynurenic acid
CA1,2,3	Hippocampal regions
LTP	Long-term potentiation
CREB	Cyclic AMP-Response-Element-Binding Protein
OLE	Oleuropein
TrkB	Tropomyosin receptor kinase B
PFC	Prefrontal cortex
NT	Neurotrophin
ADAM10	A Disintegrin and metalloproteinase domain-containing protein
BACE1	Beta-Site APP cleaving enzyme 1
PPREs	Peroxisome proliferator response elements
RXR	Retinoid X receptor
CBP	CREB-binding protein
IκBα	NFkB Inhibitor
PGC1a	Peroxisome proliferator-activated receptor gamma coactivator 1-alpha
IDE	Insulin-degrading enzyme
mtDNA	Mitochondrial DNA
TFAM	Transcription factor A, mitochondrial
VTA	Ventral tegmental area
β2nAChRs	β2-containing nicotinic acetylcholine receptors
SNpc	Substantia nigra pars compacta
PAM	Proliferative-associated microglia
MPTP	1-methyl-4-phenyl-1,2,3,6-tetrahydropyridine
GDNF	Astrocytic glial-derived neurotrophic factor
IFNγ	Interferone γ
NMDA	N-methyl-D-aspartate
HSF-1	Heat shock factor-1
GSK3β	Glycogen synthase kinase-3 beta
CDK5	Cyclin-dependent kinase 5
NRF-1	Nuclear respiratory factor-1
